# Analysis and Evaluation of the Image Preprocessing Process of a Six-Band Multispectral Camera Mounted on an Unmanned Aerial Vehicle for Winter Wheat Monitoring

**DOI:** 10.3390/s19030747

**Published:** 2019-02-12

**Authors:** Jiale Jiang, Hengbiao Zheng, Xusheng Ji, Tao Cheng, Yongchao Tian, Yan Zhu, Weixing Cao, Reza Ehsani, Xia Yao

**Affiliations:** 1 National Engineering and Technology Center for Information Agriculture, Nanjing Agricultural University, Nanjing 210095, China; jialejiang@njau.edu.cn (J.J.); 2015201019@njau.edu.cn (H.Z.); 201610111@njau.edu.cn (X.J.); tcheng@njau.edu.cn (T.C.); yctian@njau.edu.cn (Y.T.); yanzhu@njau.edu.cn (Y.Z.); caow@njau.edu.cn (W.C.); 2 Key Laboratory for Crop System Analysis and Decision Making, Ministry of Agriculture, Nanjing Agricultural University, Nanjing 210095, China; 3 Jiangsu Key Laboratory for Information Agriculture, Nanjing Agricultural University, Nanjing 210095, China; 4 Jiangsu Collaborative Innovation Center for Modern Crop Production, Nanjing Agricultural University, Nanjing 210095, China; 5 Mechanical Engineering Department, University of California-Merced, Merced, CA 95343, USA; rehsani@ucmerced.edu

**Keywords:** multispectral sensor, unmanned aerial vehicle (UAV), imagery preprocessing, leaf area index (LAI), winter wheat

## Abstract

Unmanned aerial vehicle (UAV)-based multispectral sensors have great potential in crop monitoring due to their high flexibility, high spatial resolution, and ease of operation. Image preprocessing, however, is a prerequisite to make full use of the acquired high-quality data in practical applications. Most crop monitoring studies have focused on specific procedures or applications, and there has been little attempt to examine the accuracy of the data preprocessing steps. This study focuses on the preprocessing process of a six-band multispectral camera (Mini-MCA6) mounted on UAVs. First, we have quantified and analyzed the components of sensor error, including noise, vignetting, and lens distortion. Next, different methods of spectral band registration and radiometric correction were evaluated. Then, an appropriate image preprocessing process was proposed. Finally, the applicability and potential for crop monitoring were assessed in terms of accuracy by measurement of the leaf area index (LAI) and the leaf biomass inversion under variable growth conditions during five critical growth stages of winter wheat. The results show that noise and vignetting could be effectively removed via use of correction coefficients in image processing. The widely used Brown model was suitable for lens distortion correction of a Mini-MCA6. Band registration based on ground control points (GCPs) (Root-Mean-Square Error, RMSE = 1.02 pixels) was superior to that using PixelWrench2 (PW2) software (RMSE = 1.82 pixels). For radiometric correction, the accuracy of the empirical linear correction (ELC) method was significantly higher than that of light intensity sensor correction (ILSC) method. The multispectral images that were processed using optimal correction methods were demonstrated to be reliable for estimating LAI and leaf biomass. This study provides a feasible and semi-automatic image preprocessing process for a UAV-based Mini-MCA6, which also serves as a reference for other array-type multispectral sensors. Moreover, the high-quality data generated in this study may stimulate increased interest in remote high-efficiency monitoring of crop growth status.

## 1. Introduction

Efficient monitoring of crops is the basis of precision agriculture and helps to identify, analyze, and manage crop variability within farmland [1,2]. The advantages of using unmanned aerial vehicles (UAVs) are high flexibility, high spatial resolution, and ease of operation, and their use in crop monitoring has grown rapidly in recent years, especially in precision agriculture [3,4,5].

In the past, different types of sensors, mounted on UAVs, have been used for monitoring crop growth status [6]. For example, hyperspectral and thermal infrared sensors have been used to estimate leaf area index (LAI), nitrogen content, and biomass [7,8,9]. However, the aforementioned sensors are too heavy and bulky to be carried by UAVs, and their application is also limited by their high cost and low computing efficiency [6,10]. Although consumer-level digital cameras are lightweight and affordable, they are inadequate for more in-depth and extensive research without red edge and near-infrared (NIR) band coverage regions, which are more sensitive for crop monitoring than visible bands [11]. In contrast, multispectral sensors can provide multiple spectral bands (from visible to NIR) with centimeter-level spatial resolution [6,11]. In addition, due to the advantages of being low cost, having compact dimensions, and the ability to do fast frame imaging, multispectral sensors provide an acceptable balance between affordability and usability [11,12,13].

Image preprocessing of UAV-based multispectral sensors is a basic and vital factor that influences spectral accuracy and quantitative analysis [14]. Sensor corrections are the key initial steps required for extraction of geometrically consistent at-sensor data from the raw data. This aspect of the measurement process encompasses noise correction, vignetting correction, and lens distortion correction [15]. Image noise refers to any undesirable signal produced by the sensor [16], while the purpose of noise correction is to eliminate the systematic errors of the multispectral sensors. Vignetting is a spatially dependent light intensity falloff, which leads to a reduction in radiance toward the periphery compared to the image center [17,18,19]. Lens distortion is mainly caused by the differences in magnification across a lens surface and the misalignment between the lens and the detector plane, represented by radial distortion and tangential distortion [20]. Generally, correction coefficients are calculated to correct for noise and vignetting in multispectral images [15], and the Brown model is commonly adopted for lens distortion correction [15,21,22]. In addition to the preliminary corrections, band registration is required to improve spatial consistency between bands. Although commercially available software (e.g., PhotoScan, Airsoft LLC, Russia and Tetracam PixelWrench2) are available for alignment correction, low accuracies have been found in many studies [14,23]. To improve the band misalignment, several studies have proposed the use of algorithms for the correction of misalignment errors. Laliberte et al. [14] employed a Local Weighted Mean Transform (LWMT) method [24] to detect edges; however, only a local translation effect was considered. Radhadevi et al. [25] used a photogrammetric technique to remove unaccounted-for misregistration residuals; however, it was only suitable for small, flat areas. Turner et al. [23] developed new algorithms based on the Scale Invariant Feature Transform (SIFT); however, an error assessment was not undertaken. After band registration, the multispectral images still contain the digital number (DN) values. To convert the DN values into spectral reflectance, radiometric correction is required. Two radiometric correction methods, vicarious correction and preflight correction, are often employed for UAV-based multispectral sensors [23]. As one of the most commonly used vicarious correction methods, the empirical linear calibration (ELC) method depends on a regression analysis of spectral data from images and the real measured values [23], and it has been applied in a variety of crop-monitoring studies [10,26,27]. The preflight calibration used laboratory-calibrated parameters (e.g., calibration coefficients) to characterize the sensor [28]. The two types of radiometric calibration methods have been used in different UAV-based sensors; however, there has been a lack of comparative studies to access accuracy and applicability.

Image preprocessing of UAV-based multispectral sensors basically involves five steps, namely noise correction, vignetting correction, lens distortion correction, band registration, and radiometric correction. Some studies have described procedures for sensor correction [15], and some have attempted to adopt different methods for band registration [14,23,29] or radiometric calibration [30]. However, a quantitative study on the accuracy of the complete preprocessing process has not been performed. Moreover, the performance of calibration methods varies for different UAV onboard sensors. There is, therefore, a timely need in crop monitoring research to analyze each correction procedure for a specific sensor and evaluate the applicability of different calibration methods in crop monitoring.

In this study, a typical six-band multispectral sensor (Mini-MCA6), which has been widely used in crop monitoring [31,32,33,34,35], was adopted to permit an evaluation of the image preprocessing steps. The objectives of the study were as follows: (1) to analyze the components of sensor error or data modification within the UAV-based multispectral sensor; (2) to compare the accuracy of intrinsic (software-based) and extrinsic (ground control point-based) methods for band registration; (3) to assess the performance of preflight calibration and vicarious calibration, i.e., ELC methods, for radiometric correction of a narrowband multispectral sensor; and (4) to evaluate the applicability and potential of image preprocessing for crop monitoring purposes. The anticipated results would provide guidance on how to select a robust fit-for-purpose method for multispectral imagery preprocessing, and the suggested methods, integrated on ENVI/IDL platforms, provide a semi-automated image preprocessing process of a six-band multispectral camera.

## 2. Materials and Methods

### 2.1. Imaging Sensor and UAV System

**Figure 1:**
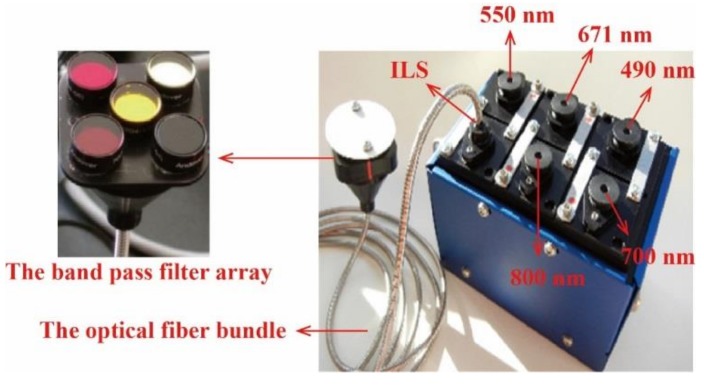
A photo of the Tetracam Mini-MCA6. ILS, incident light sensor.

A six-band multispectral camera (Mini-MCA6 Tetracam, Inc., Chatsworth, CA, USA) was used. The Mini-MCA6 is a miniature array camera with five band channels and an incident light sensor (ILS), which contains a band pass filter and an optical fiber (Figure 1). The basic performance parameters of the Mini-MCA6 are summarized in Table 1.

**Figure 2:**
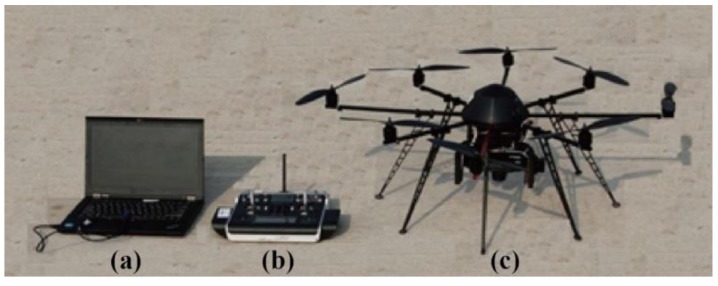
A photo of the UAV system equipped with (**a**) a ThinkPad laptop; (**b**) a Graupner MC-32 control module; and (**c**) an ARF-MikroKopter UAV.

The multispectral camera was mounted on an ARF-MikroKopter UAV (Mikrokopter Inc., Moormerland, Germany), which had eight rotors. The specifications of the UAV are listed in Table 2. The UAV system was equipped with a MC-32 remote control module and a ThinkPad laptop, as shown in Figure 2.

### 2.2. Image-Preprocessing Methods

#### 2.2.1. Noise Correction

Noise correction was performed to correct the systematic error of the multispectral sensors in the first step of multispectral image preprocessing. Given that the raw digital number (*DN_raw_*) of each pixel is the sum of a noise component (*DN_noise_*) and a radiance component (*DN_rad_*), identifying the characteristic components of the noise component is the key to extracting the radiance component [15].

(1) $$DN_{rad}=DN_{raw}-DN_{noise}$$

To appropriately access the contribution of the image noise, the multispectral camera was kept in a fully enclosed black box, which removed the radiance component as a result of the physical isolation of the sensor. The setup would then generate dark offset imagery to characterize the distribution of image noise on a per-pixel basis. For each band channel of the Mini-MCA6, a sensor-specific database of dark offset imagery was conducted at the same three exposure levels (1.0, 1.5, and 2.0 ms) as the field test. According to a trial-and-error method, the number of dark offset images for each exposure time was set as 100 to balance correction accuracy and calculation efficiency. The noise component of each pixel was calculated from the average of the 100 images and stored as separate images [15]. Then, the image noise could be corrected based on Equation (1).

#### 2.2.2. Vignetting Correction

**Figure 3:**
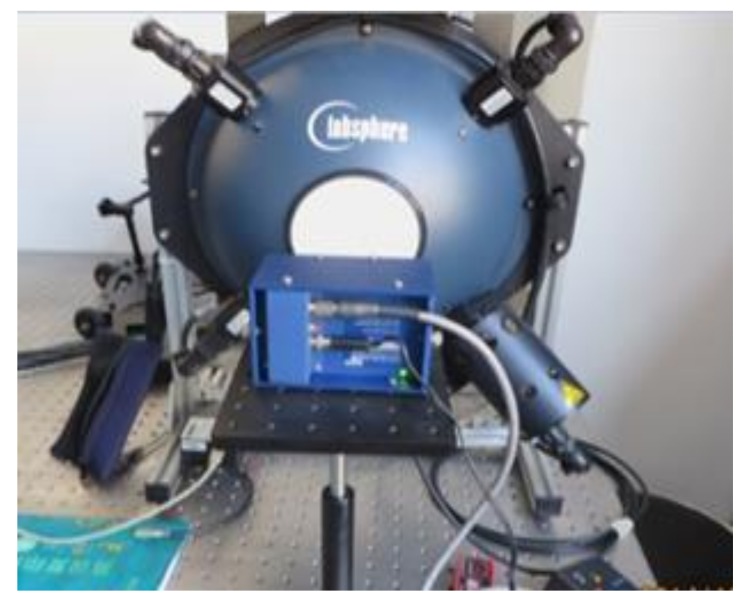
A photo of the uniform source system (CSTM-USS-1200C; Labsphere, Inc., North Sutton, NH, USA).

Due to the non-uniformity of the optical lens, the brightness or saturation of an image would decrease toward the periphery compared to the image center, namely vignetting. We used the uniform source system (CSTM-USS-1200C; Labsphere, Inc., North Sutton, NH, USA), as shown in Figure 3, to create vignetting images at three exposure levels (1.0, 1.5, and 2.0 ms). To maximize the noise reduction potential, 100 vignetting images per exposure level were averaged as vignetting samples (*V_a_*) for each band channel [15]. Due to the increasing signal-to-noise ratio (SNR) from the center to the edge of images, the periphery of the vignetting samples had high uncertainty [15]. To ensure the data’s quality, the top 5% of DN values in *V_a_* were averaged as *V_b_*. The correction coefficient of vignetting (*V_c_*) for each channel was obtained from:

(2) $$V_{c}=V_{a}/V_{b}.$$

#### 2.2.3. Lens Distortion Correction

Lens distortion was corrected using the Brown model [21]. The lens distortion correction parameters for each channel were measured using a program supplied by the Beijing Spatial Information Technology Co., Ltd., and the coefficients are given in Table 3. Due to the different distortions, the five channels should be corrected separately based on the appropriate correction coefficients:

(3) $$\begin{array}{c} \Delta x=(x-x_{0})(k_{1}r^{2}+k_{2}r^{4})+p_{1}[r^{2}+2{\left(x-x_{0}\right)}^{2}],\\ +2p_{2}(x-x_{0})(y-y_{0})+\alpha (x-x_{0})+\beta (y-y_{0}) \end{array}$$

(4) $$\Delta y=(y-y_{0})(k_{1}r^{2}+k_{2}r^{4})+p_{2}[r^{2}+2{\left(y-y_{0}\right)}^{2}]+2p_{1}(x-x_{0})(y-y_{0}),$$

(5) $$r=\sqrt{{\left(x-x_{0}\right)}^{2}+{\left(y-y_{0}\right)}^{2}},$$

where *∆x* and *∆y* are the image correction values along the horizontal and vertical coordinates, respectively, *x* and *y* are the coordinates of the image point, *x_0_* and *y_0_* represent the main image point, *k_i_* and *p_i_* (*I* = 1, 2) are the coefficients of radial distortion and decentering distortion, respectively, *α* is the non-square scaling factor, and *β* is the non-orthogonal distortion factor.

#### 2.2.4. Band Registration

**Figure 4:**
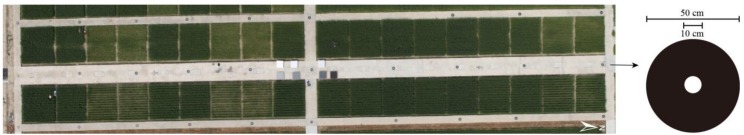
The distribution and the size of the ground control points (GCPs).

The lenses of the multispectral camera were arranged in two rows as depicted in Figure 1. The misalignment of these lenses leads to displacement between the images from each channel. To eliminate image displacement, band registration was performed. Two methods for band registration were used. One was via the Tetracam PixelWrench2 (PW2) software, which was embedded in the multispectral camera [35]. The other was based on the ground control point-based (GCP-based) method. To distinguish GCPs easily, the GCPs were painted on the road as black annuluses with inner and outer diameters of 10 cm and 50 cm, respectively (Figure 4). The geographic coordinates of GCPs were measured by RTK-GPS (X900 GNSS, Huace., Beijing, China). The image of MCA-0 was used as the reference image and the images of the other channels were registered with the reference image via the image registration tool (i.e., “select GCPs: image to image”) in ENVI 5.2.

#### 2.2.5. Radiometric Correction

Radiation correction was necessary to eliminate distortion information in the radiance data. Two methods for radiation correction were employed: the ELC method [36] and the light intensity sensor calibration (ILSC) method.

**Figure 5:**
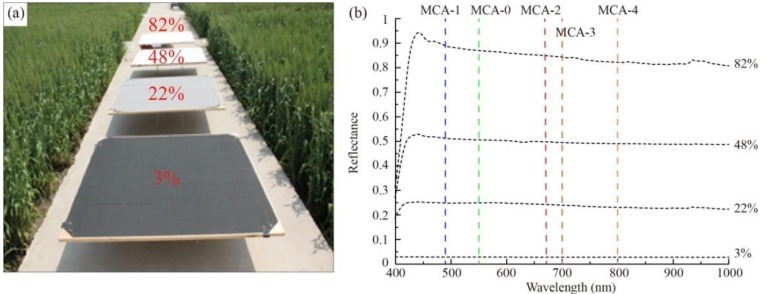
The calibration canvas with different reflectance values (**a**) and the corresponding spectral signatures (**b**).

The ELC is a typical vicarious correction method based on a regression analysis of spectral data from images and the real measured values. The standard data were measured by an analytical spectral device (ASD) FieldSpec Pro spectrometer (Analytical Spectral Devices, Boulder, CO, USA) from four pieces of the calibration canvas with different reflectance values of 3%, 22%, 48%, and 82% (Figure 5). As shown in Figure 5b, the spectral signatures of the calibration canvas were stable and in the range of 490–800 nm, including all the wavelengths of the band channels (see Table 1).

**Figure 6:**
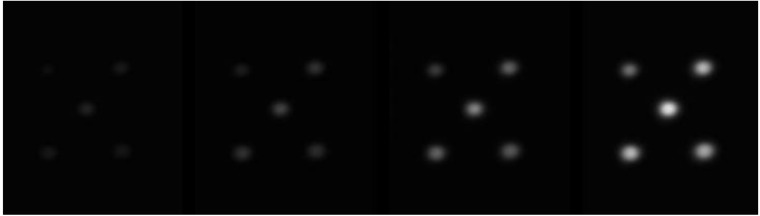
The correction file from the light intensity sensor.

The principle of ILSC is to multiply the ratio of the downward intensity to the reflected light intensity by the correction factors. The information of downward intensity was captured through the optical fiber (see Figure 1) and stored in the ILS. The corresponding correction file derived from the light intensity sensor can be uploaded into the PW2 software as depicted in Figure 6.

### 2.3. Winter Wheat Case Study

The study area was located in the town of Baipu, which is in the city of Rugao, Jiangsu Province, China (120°45’E, 32°16’N). Two winter wheat (*Triticum aestivum* L.) field experiments were designed involving different nitrogen (N) application rates, planting densities (D), and varieties (V) in two growing seasons. Details of the field experiments are given in Table 4. To avoid the uncertainty from image mosaicking, the multispectral images were captured at an altitude of 150 m, resulting in covering all 36 field plots within a single image and a spatial resolution of 8.125 cm. All flights were carried out between 11:00 and 13:00 in stable ambient light conditions together with field measurement of the LAI and leaf biomass on the same day.

The LAI was measured as follows. For each plot, 30 wheat stems were considered as one sample, and the number of stems in one meter (*B*) was counted manually. The green leaf area was scanned using the LI-3000 (LI-COR Inc., Lincoln, NE, USA). The LAI of the population was calculated by:

(6) $$LAI=1/D\times B\times A/C\times 10^{-4},$$

where *D* was the distance between two rows of wheat, and *A* and *C* were the leaf area and number of stems, respectively.

The leaf biomass was measured as follows: For each plot, 30 hills of plants were cut above the ground surface. All green leaves and panicles were separated from the stems. All components were oven-dried at 105 °C for 30 min and then at 80 °C for about 24 h until a constant weight was obtained.

To analyze the performance of the different image-preprocessing methods, the data of Experiment 1 were analyzed. Based on the results, an appropriate image preprocessing process was proposed for the UAV-based multispectral sensor. To evaluate the feasibility and stability of the optimal image-preprocessing methods for crop monitoring, the estimation models of LAI and leaf biomass for winter wheat were validated with the data from Experiment 2. Eight commonly used vegetation indexes (VIs) were selected for the estimation of LAI and leaf biomass as indicated in Table 5.

## 3. Results

### 3.1. Sensor Error Analysis

#### 3.1.1. Noise Correction Factor for Each Band

The average noise images for the five channels of the Mini-MCA6 and the variation of these noise values (*DN_noise_*) with the exposure time are presented in Table 6. For each band, the noise images were not exactly the same under different exposure times, and the values did not change regularly as the exposure time increased. For the same exposure time, the *DN_noise_* varied from band to band within the range 0–15. Compared to the other four bands, MCA-2 had the lowest noise value. According to the *DN_noise_* for each band at specific exposure times in Table 6, the systematic error of the Mini-MCA6 caused by noise would be eliminated by Equation (1).

#### 3.1.2. Vignetting Correction for Each Band

As shown in Table 7, the images for the vignetting correction factor for each band were not symmetric circular distributions. For the same exposure time, the factor images and the offsets of the circle center for the five bands were different. For the same band, the vignetting correction factors were different with different exposure times. The distributions of *V_c_* in the middle row of the images for each channel were accordingly listed in Table 7. The range of *V_c_* was from 1 to 1.5 for MCA-0, MCA-1, MCA-3, and MCA-4, while the value of *V_c_* for MCA-2 was between 1 and 2.4. It is obvious that the peak of *V_c_* was not in the middle for all channels.

**Figure 7:**
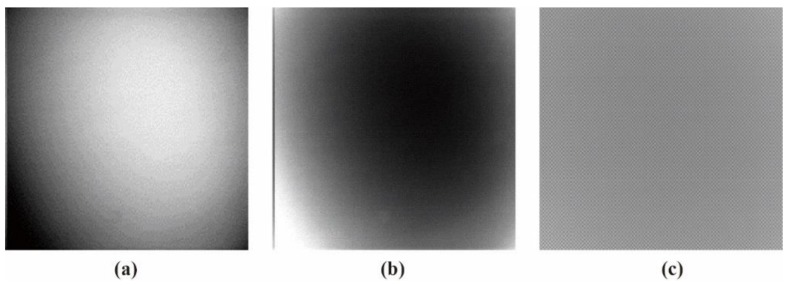
A comparison of the MCA-0 images (**a**) before and (**c**) after vignetting correction based on the correction factor image (**b**).

Based on the images of correction factors, the vignetting could be eliminated. Taking the correction result of MCA-0 as an example, the DN values of the corrected image were distributed evenly after correction (Figure 7). The correction results for other band channels of the Mini-MCA6 were similar to that of MCA-0.

#### 3.1.3. Brown Model for Lens Distortion Correction

**Figure 8:**
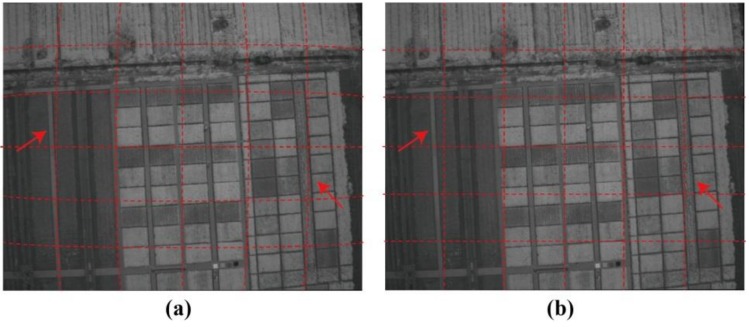
A comparison of the MCA-4 images (**a**) before and (**b**) after lens distortion correction at the booting stage.

Taking the MAC-4 images at the booting stage as an example, Figure 8 shows the results before and after correction of lens distortion via the Brown model. The correction results for other images of the Mini-MCA6 were found to be similar. In a visual assessment, the lens distortion was effectively eliminated based on processing based on the Brown model.

### 3.2. Comparison of PW2-Based and GCP-Based Methods for Band Registration

**Figure 9:**
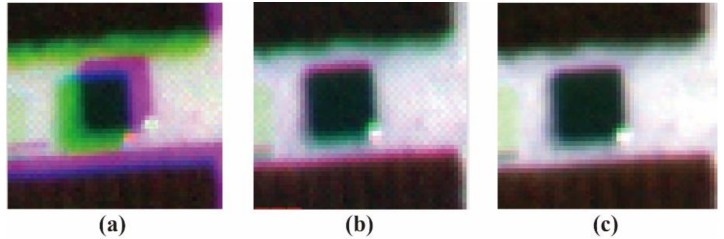
Mini-MCA6 multispectral images from different band registration methods. (**a**) Original image stacking; (**b**) PW2-based method; and (**c**) GCP-based method.

**Figure 10:**
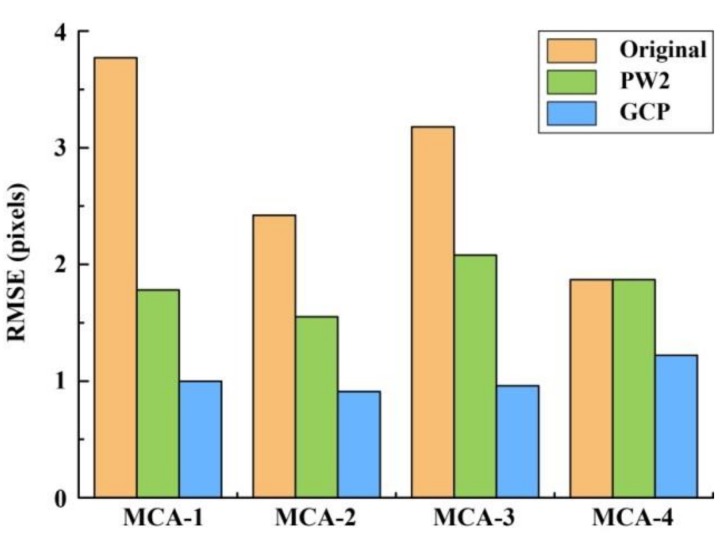
The root-mean-square error (RMSE) of mismatched pixels between each slave and the main channel for different band registration methods.

Figure 9 shows one example of (a) the original, (b) the PW2-based, and (c) the GCP-based results. A visual inspection of the data revealed that pixel displacement of different bands was eliminated by both the PW2-based and GCP-based methods after band registration between the reference channel (MCA-0) and the other four bands. Compared to the multispectral image from the PW2-based method (see Figure 9b), the boundaries of features in the corrected image from the GCP-based method (see Figure 9c) gave a better match between each band. The statistical results also indicated that the GCP-based method was superior to the PW2-based method (Figure 10). Although the root-mean-square errors (RMSEs) of mismatched pixels for MCA-1, MCA-2, and MCA-3 decreased after PW2-based registration, the match between MAC-4 and the master channel was not improved. On the contrary, the RMSEs for all slaves could be reduced to about one pixel by the GCP-based method.

### 3.3. Comparison of the ILSC and ELC Methods for Radiometric Correction

**Figure 11:**
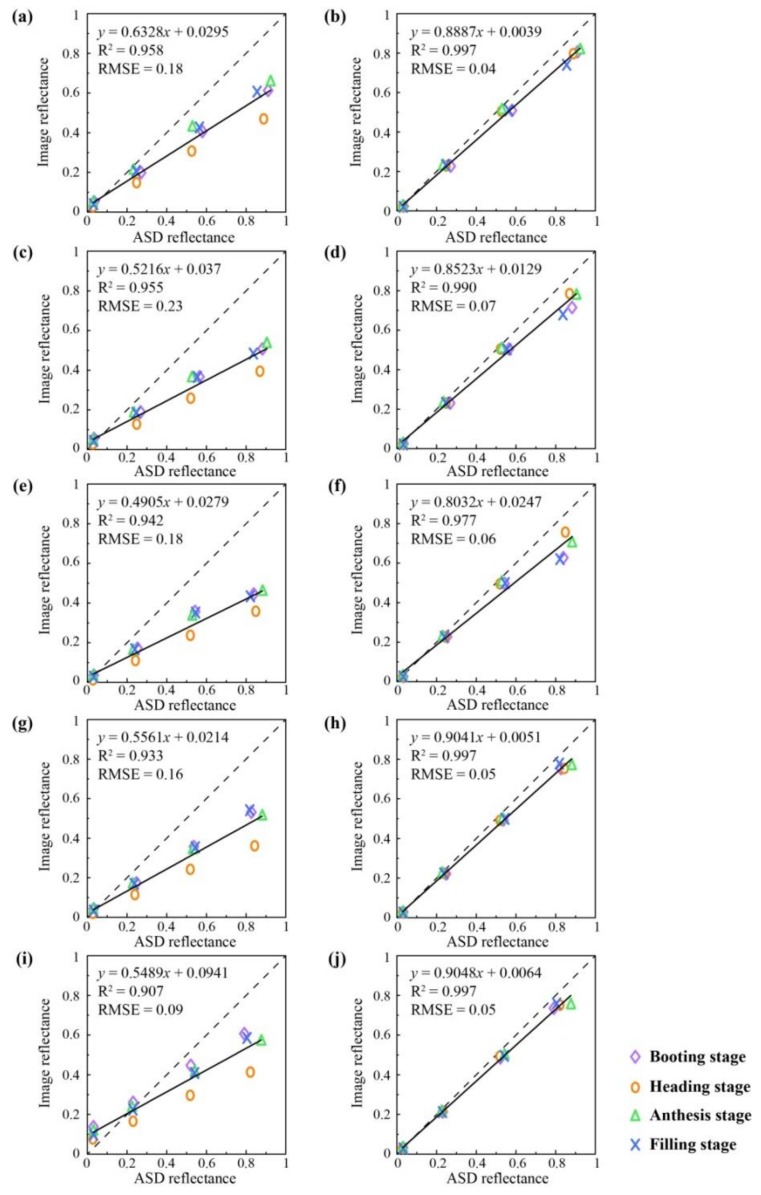
Comparison of the analytical spectral device (ASD) reflectance with the calibrated values for five bands at four growth stages with the light intensity sensor correction (ILSC) and empirical linear correction (ELC) radiometric correction methods. Panels (**a**–**b**), (**c**–**d**), (**e**–**f**), (**g**–**h**), and (**i**–**j**) are the plots for the MAC-1, MCA-0, MAC-2, MAC-3, and MAC-4, respectively. The two columns from left to right correspond to the radiometric correction methods ILSC and ELC, respectively.

The reflectances of the four correction canvases from the ASD spectrometer were used to evaluate the performances of the ILSC and ELC methods. As shown in Figure 11, the ELC method performed significantly better than the ILSC method. For the ILSC method, the results of the heading stage were generally underestimated. Compared to the ILSC method, the RMSEs of the ELC method were reduced by an average of 65.4% for all bands, and the largest improvement was in MCA-1, with RMSE values ranging from 0.18 to 0.04.

### 3.4. Estimation of LAI and Leaf Biomass

**Figure 12:**
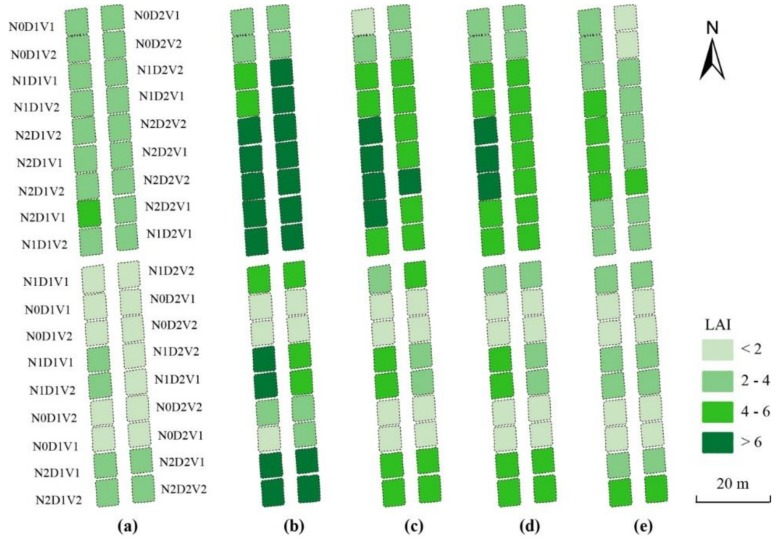
Leaf area index (LAI) mappings using the relationship between MTVI_2_ and LAI at different growth stages: (**a**) jointing stage; (**b**) booting stage; (**c**) heading stage; (**d**) anthesis stage; and (**e**) filling stage. N is nitrogen rate (N0 = 0, N1 = 150, N2 = 300 kg/ha). D is row spacing (D1 = 25, D2 = 40 cm). V represents the wheat varieties Shengxuan 6 (V1) and Yangmai 18 (V2).

**Figure 13:**
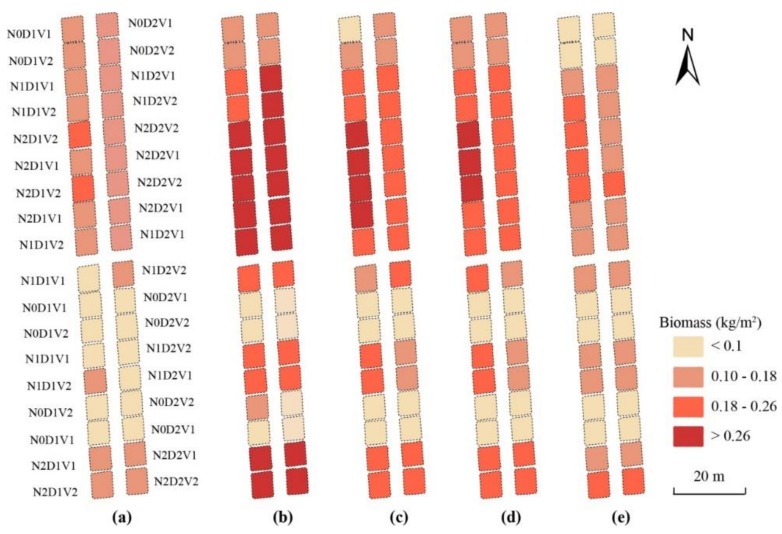
Leaf biomass mappings using the relationship between MTVI_2_ and leaf biomass at different growth stages: (**a**) jointing stage; (**b**) booting stage; (**c**) heading stage; (**d**) anthesis stage; and (**e**) filling stage. N is nitrogen rate (N0 = 0, N1 = 150, N2 = 300 kg/ha). D is row spacing (D1 = 25, D2 = 40 cm). V represents the wheat varieties Shengxuan 6 (V1) and Yangmai 18 (V2).

To evaluate the feasibility and stability of the optimal image-preprocessing methods (noise correction, vignetting correction, lens distortion correction with the Brown model, GCP-based band registration, and radiometric correction with the ELC method), the LAI and the leaf biomass of winter wheat were estimated for 2014–2015. As shown in Table 8, the VIs extracted from the corrected multispectral images were of satisfactory performance for the estimations of both LAI (R^2^ > 0.76 and RMSE < 1.13) and leaf biomass (R^2^ > 0.73 and RMSE ≤ 0.051). The highest accuracies for the LAI and the leaf biomass estimations were produced by MTVI_2_ (modified triangular vegetation index). Based on the MTVI_2_ images at different growth stages, the mappings of LAI and leaf biomass were generated as shown in Figure 12 and Figure 13, respectively. At the same growth stage, the LAI and leaf biomass of winter wheat increased with a rise in plant density and nitrogen level. Under the same treatment regime, the LAI and the leaf biomass increased at an early stage and then decreased. The results were reasonable and consistent with a visual inspection.

## 4. Discussion

### 4.1. Correction of Noise, Vignetting, and Lens Distortion

Noise, vignetting, and lens distortion generated by sensors are important factors that dictate the quality of multispectral images from the Mini-MCA6. In this study, we identified and quantified those components of sensor error for each spectral band at the different growth stages of wheat.

To acquire uncontaminated information, noise images were generated for the noise correction in this study. The results indicated that the noise components of the different spectral bands at the different exposure times should be corrected separately and specifically, which is consistent with the results of an earlier study [15]. Additionally, due to the different observation conditions, real-time corrections were required for multispectral images acquired at the different growth stages of winter wheat.

For the vignetting correction, Laliberte et al. [14] used the correction files of the multispectral camera in PW2. However, the vignetting correction factors were not fixed, but gradually changed in a circular ring (Table 7). Therefore, it would be more appropriate to determine the vignetting correction factors of each spectral band on the exposure time setting of the UAV-based sensors.

The Brown model has been frequently used for lens distortion correction in digital photography and multispectral cameras [15]. In this study, the robustness of the Brown model was verified, and the results showed that the model effectively reduced the lens distortion of the Mini-MCA6. Since the performance of the Brown model was evaluated by a visual assessment, a quantitative comparison will be performed in future work for the purpose of in-depth discussion.

### 4.2. Selection of Appropriate Methods for Spectral Band Registration and Radiometric Correction

Given that the UAV-based multispectral images have a significant misregistration error between different spectral bands, various methods have been applied to spectral band registration. However, assessment of accuracy has rarely been discussed. In this study, both intrinsic (PW2-based) and extrinsic (GCP-based) methods were evaluated. Although the PW2-based method was automated, our results showed that misregistration errors could not be effectively eliminated (an average RMSE of 1.82 pixels was obtained), which was consistent with earlier work [14]. Another highly automated method was proposed by Turner et al. [23], who used the SIFT algorithm to find feature points in the image for band registration, and obtained an average accuracy of 1.78 pixels. However, the feature points of crops might not be precisely detected by such feature-based methods, especially for cropland with indistinct boundaries. In contrast, the GCP-based method was performed artificially for band registration and produced better results, with an average RMSE of 1.02 pixels. It is noted that it was difficult to distinguish GCPs when the flight height was higher than 400 m [29]. Given the above, the GCP-based band registration method is recommended for the acquisition of high-resolution Mini-MCA6 images in crop monitoring.

The purpose of radiometric correction is to convert the DN values of each pixel to a reflectance value in the image. Two methods (ILSC and ELC) were used for preprocessing the Mini-MCA6 images in this study. Compared with the reflectance spectra acquired by the ASD spectrometer, the performance of the ELC method was better than that of the ILSC method. This might be because the correction files of the ILSC method were produced directly at a height of 150 meters and the light and solar radiation intensities would have been different when the height of the UAV was changed. In the case of the ELC method, the correction coefficients were determined by the use of real measured values. Therefore, the ELC method is considered to be appropriate and fit-for-purpose for radiometric correction of the Mini-MCA6.

### 4.3. Application of Image Preprocessing in Crop Monitoring

Based on the validation and evaluation of the above methods, image preprocessing of UAV-based Mini-MCA6 data should consist of the following: (1) noise correction; (2) vignetting correction; (3) lens distortion correction via the Brown model; (4) GCP-based band registration; and (5) radiometric correction with the ELC method. To evaluate the feasibility and stability of the image preprocessing process, the LAI and leaf biomass of winter wheat were estimated based on the post processed Mini-MCA6 images. The results clearly demonstrated the applicability and potential for image preprocessing in winter wheat monitoring. Moreover, the utility and practicality of those methods have been increased by developing all of the procedures on ENVI/IDL platforms.

The present study was based on field plot experiments of winter wheat. Although the field experiments were conducted over two years with specific wheat varieties, planting densities, and N application rates, the spatial heterogeneity and spectral differences of plots had some regularity. Therefore, other crops grown under different conditions should be used to test the applicability of the proposed image preprocessing process. The proposed image preprocessing process can also serve as a reference for other array-type multispectral sensors.

## 5. Conclusions

In this study, an analysis and evaluation of the image preprocessing of a UAV-based multispectral sensor, which included noise correction, vignetting correction, lens distortion correction, spectral band registration, and radiometric correction, has been performed. The results showed that the difference (or ratio) between the noise (or vignetting) images of the Mini-MCA6 and the raw images could effectively reduce the error caused by noise (or vignetting). The Brown model proved to be highly versatile and robust for lens distortion correction. For spectral band registration, the GCP-based method is recommended if the GCPs can be clearly observed in high-resolution images. Regarding radiometric correction, the ELC method performed better than ILSC; thus, the ELC method is appropriate for narrowband multispectral images.

In practical use, the proposed procedures can be processed on the ENVI/IDL platforms. Based on the field plot experiments of winter wheat, the LAI and leaf biomass at four critical growth stages were estimated to evaluate the feasibility of the image-preprocessing process. The results demonstrated that the image-preprocessing process for a UAV-based Mini-MCA6 was reliable for winter wheat monitoring, and also serves as a reference for other array-type multispectral sensors. The proposed image-preprocessing process should be extended to diverse farmlands for other crop monitoring investigations.

## Figures and Tables

**Table 1:** The performance parameters of the Tetracam Mini-MCA6.

Channel	Central Wavelength (nm)	Bandwidth (nm)	Exposure Proportion (%)
Master (MCA-0)	550	10	70
Slave 1 (MCA-1)	490	10	120
Slave 2 (MCA-2)	671	10	100
Slave 3 (MCA-3)	700	10	100
Slave 4 (MCA-4)	800	10	80
Incident light sensor (ILS)			130

**Table 2:** The specifications of the parameters for the ARF-MikroKopter unmanned aerial vehicle (UAV).

Parameter	Value
Weight	2050 g
Size	73 (width) × 73 (length) × 36 (height) cm
Battery 4s/5000	520 g
Maximum payload	2500 g
Flight duration	8–41 min
Temperature range	−5–35 °C

**Table 3:** The lens distortion correction coefficients for the Mini-MCA6.

Correction Coefficients		Channel 1	Channel 2	Channel 3	Channel 4	Channel 5
Main image point	*x*_0_	459.2210	457.1416	459.5102	458.7621	447.4720
	*y*_0_	553.2878	553.6993	553.4641	558.4854	554.4125
Radial distortion	*k*_1_	5.943 × 10^−8^	5.396 × 10^−8^	5.836 × 10^−8^	4.322 × 10^−8^	5.635 × 10^−8^
	*k*_2_	−7.420 × 10^−15^	3.817 × 10^−15^	1.007 × 10^−14^	7.428 × 10^−15^	5.563 × 10^−16^
Decentering distortion	*p*_1_	9.080 × 10^−6^	9.836 × 10^−6^	9.064 × 10^−6^	1.015 × 10^−5^	9.353 × 10^−6^
	*p_2_*	3.876 × 10^−7^	2.110 × 10^−7^	5.372 × 10^−7^	1.763 × 10^−8^	1.307 × 10^−7^
Non-square scaling	*α*	9.220 × 10^−3^	8.82 × 10^−3^	1.012 × 10^−2^	9.240 × 10^−3^	1.028 × 10^−2^
Non-orthogonal distortion	*β*	9.554 × 10^−3^	8.941 × 10^−3^	9.234 × 10^−3^	8.603 × 10^−3^	9.920 × 10^−3^

**Table 4:** Experimental design and sampling dates for the two growing seasons.

Experiment	Experiment 1	Experiment 2
Year	2013–2014	2014–2015
Wheat cultivar	Ningmai 13, Xumai 30	Shengxuan 6, Yangmai 18
Row spacing (cm)	25	25, 40
N application rates (kg/ha)	0, 75, 150, 225, 300	0, 150, 300
Sampling dates	April 9/15/23, 2014 May 6, 2014	March 13, 2015 April 9/17/24, 2015 May 9, 2015

**Table 5:** The formulas for the vegetation indexes (VIs).

VIs^1^	Algorithm	Reference
DVI	R_800_ – R_700_	[37]
NDVI	(R_800_ – R_700_)/(R_800_ + R_700_)	[38]
GNDVI	(R_800_ – R_550_)/(R_800_ + R_550_)	[39]
RVI	R_800_ / R_700_	[40]
SAVI	1.5 × (R_800_ – R_700_)/(R_800_ + R_700_ + 0.5)	[41]
MTVI_2_	1.5 × (1.2 × (R_800_ – R_550_) – 2.5 × (R_700_ – R_550_))/(2 × (R_800_ + 1)^2^ – (6 × R_800_ – 5 × R_700_)^0.5^ – 0.5)^0.5^	[42]
EVI	2.5 × (R_800_ – R_700_)/(1 + R_800_ + 6 × R_700_ – 7.5 × R_490_)	[43]
GBNDVI	(R_800_ – (R_550_ + R_490_))/(R_800_ + R_550_ + R_490_)	[44]

**^1^** DVI = Difference Vegetation Index, NDVI = Normalized Difference Vegetation Index, GNDVI = Green Normalized Difference Vegetation Index, RVI = Ratio Vegetation Index, SAVI = Soil Adjusted Vegetation Index, MTVI = Modified Triangular Vegetation Index, EVI = Enhanced Vegetation Index, GBNDVI = Green-Blue Normalized Difference Vegetation Index.

**Table 6:** The average noise images for the five channels of the Mini-MCA6 at different exposure times.

Band	Exposure Time			
	1.0 ms	1.5 ms	2.0 ms	
MCA-0 (550 nm)	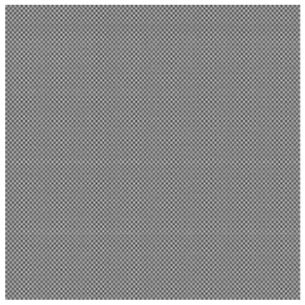	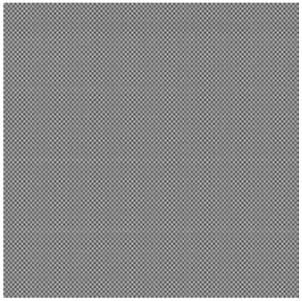	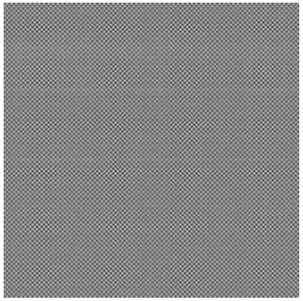	
MCA-1 (490 nm)	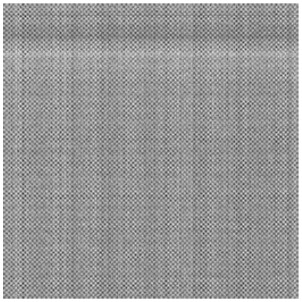	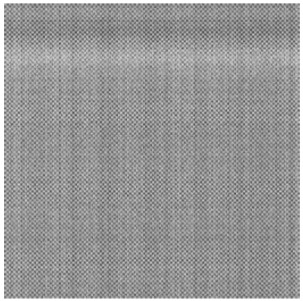	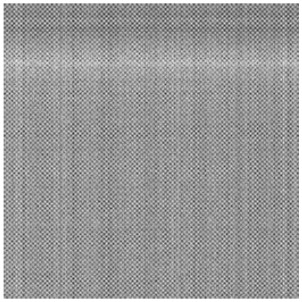	
MCA-2 (671 nm)	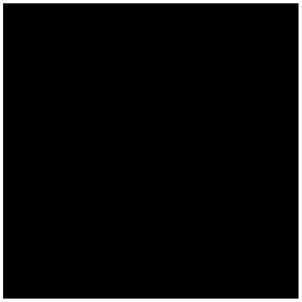	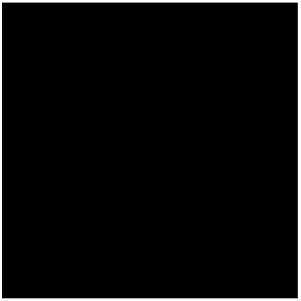	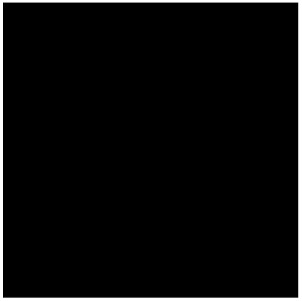	
MCA-3 (700 nm)	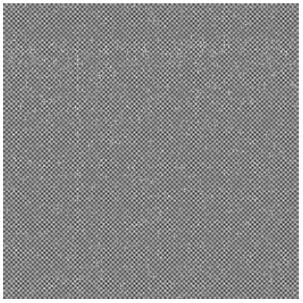	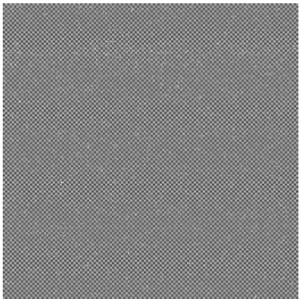	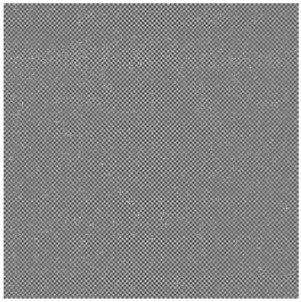	
MCA-4 (800 nm)	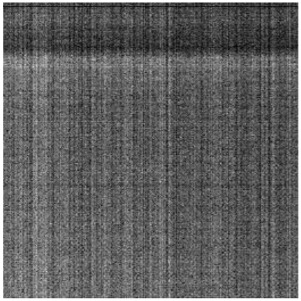	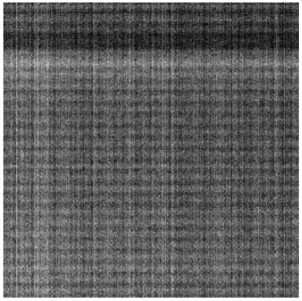	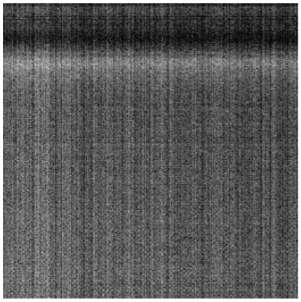	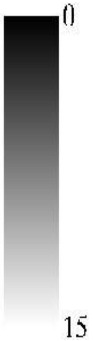

**Table 7:** The images for the vignetting correction factor (*Vc*) for the five channels of the Mini-MCA6 at different exposure times.

Band	Exposure Time			*V_C_* with the Column Number for the Middle Row ^1^
	1.0 ms	1.5 ms	2.0 ms	
MCA-0 (550 nm)	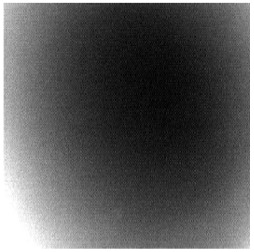	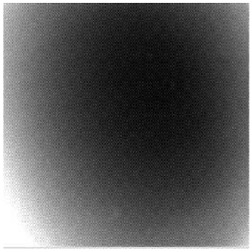	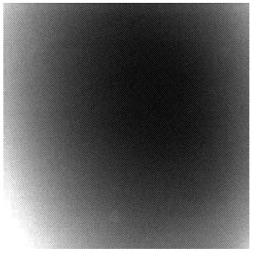	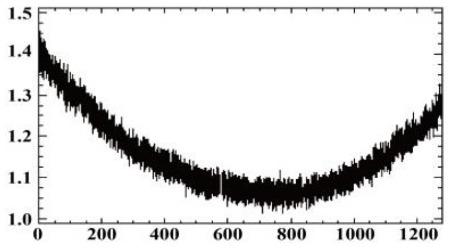
MCA-1 (490 nm)	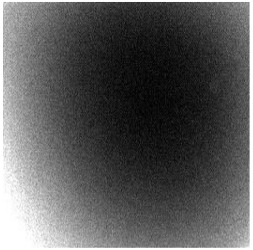	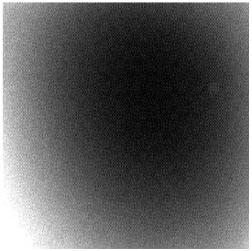	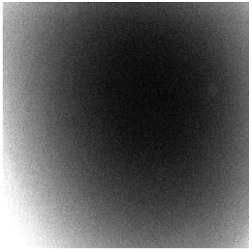	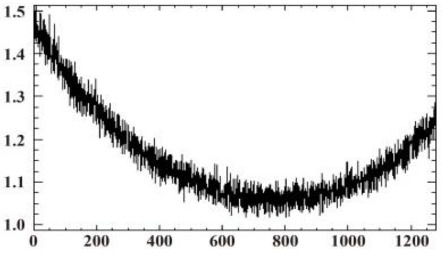
MCA-2 (671 nm)	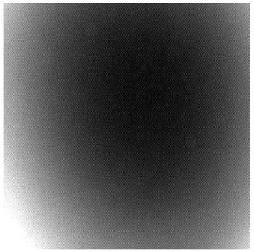	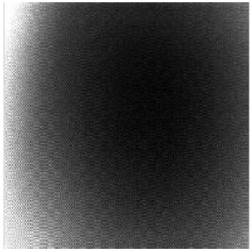	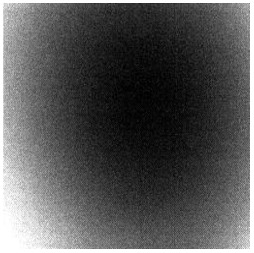	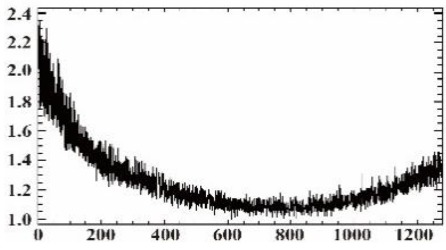
MCA-3 (700 nm)	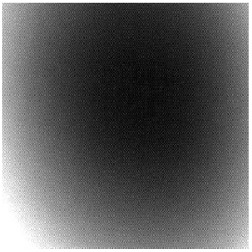	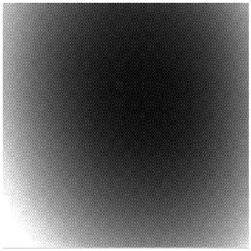	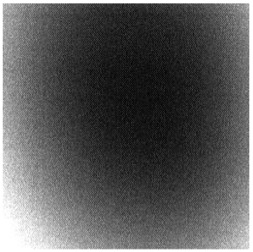	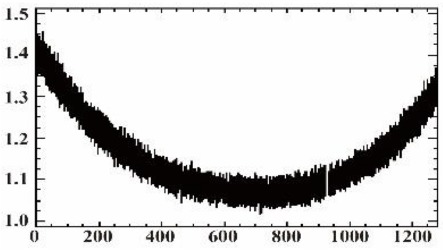
MCA-4 (800 nm)	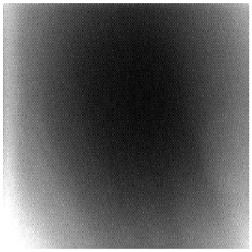	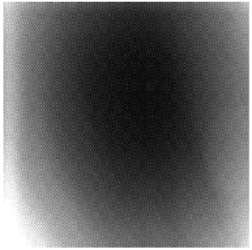	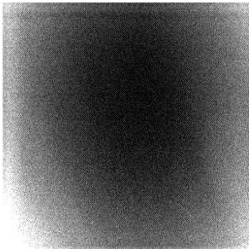	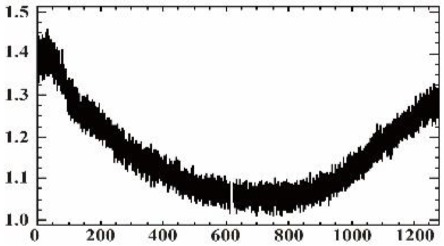

**^1^** The horizontal and vertical coordinates of the plots are column number and *V_c_*, respectively.

**Table 8:** Statistical assessments for the estimation of leaf area index (LAI) and leaf biomass with vegetation indices (VIs) for the Mini-MCA6 images. R^2^ is the determination coefficient, and RMSE is the root-mean-square error. Values in bold indicate the best accuracy (column basis).

VIs	LAI			Leaf Biomass		
	Equation	R^2^	RMSE	Equation	R^2^	RMSE
DVI	y = 0.4862e^5.6717x^	0.808	1.131	y = 0.029e^4.8811x^	0.757	0.050
NDVI	y = 0.0818e^4.9699x^	0.790	1.086	y = 0.0062e^4.2863x^	0.744	0.045
GNDVI	y = 0.0248e^6.3034x^	0.778	1.195	y = 0.0021e^5.4789x^	0.744	0.048
RVI	y = 0.707e^0.1917x^	0.833	1.130	y = 0.0393e^0.1671x^	0.801	0.044
SAVI	y = 0.1188e^5.3964x^	0.839	0.955	y = 0.0086e^4.6535x^	0.790	0.044
MTVI_2_	y = 0.5147e^3.837x^	**0.855**	**0.870**	y = 0.0303e^3.3102x^	**0.806**	**0.041**
EVI	y = 0.2409e^3.8655x^	0.783	1.127	y = 0.0159e^3.3185x^	0.731	0.050
GBNDVI	y = 0.1047e^4.7458x^	0.765	1.294	y = 0.0074e^4.1367x^	0.736	0.051
